# Interactive effects of tropospheric ozone and blast disease (*Magnaporthe oryzae*) on different rice genotypes

**DOI:** 10.1007/s11356-022-19282-z

**Published:** 2022-02-24

**Authors:** Muhammad Shahedul Alam, Angeline Wanjiku Maina, Yanru Feng, Lin-Bo Wu, Michael Frei

**Affiliations:** 1 Department of Agronomy and Crop Physiology, Institute for Agronomy and Plant Breeding, Justus-Liebig University Giessen, 35390 Giessen, Germany; 2 INRES Plant Pathology, University of Bonn, 53115 Bonn, Germany; 3 Institute for Crop Science and Resource Conservation (INRES), Crop Science, University of Bonn, 53115 Bonn, Germany

**Keywords:** Air pollution, Breeding, Cereals, Food security, Global change, Plant pathogens

## Abstract

Rising tropospheric ozone concentrations can cause rice yield losses and necessitate the breeding of ozone-tolerant rice varieties. However, ozone tolerance should not compromise the resistance to important biotic stresses such as the rice blast disease. Therefore, we investigated the interactive effects of ozone and rice blast disease on nine different rice varieties in an experiment testing an ozone treatment, blast inoculation, and their interaction. Plants were exposed to an ozone concentration of 100 ppb for 7 h per day or ambient air throughout the growth period. Half of the plants were simultaneously infected with rice blast inoculum. Grain yield was significantly reduced in the blast treatment (17%) and ozone treatment (37%), while the combination of both stresses did not further decrease grain yields compared to ozone alone. Similar trends occurred for physiological traits such as vegetation indices, normalized difference vegetation index (NDVI), photochemical reflectance index (PRI), Lichtenthaler index 2 (Lic2), and anthocyanin reflectance index 1 (ARI1), as well as stomatal conductance and lipid peroxidation. Ozone exposure mitigated the formation of visible blast symptoms, while blast inoculation did not significantly affect visible ozone symptoms. Although different genotypes showed contrasting responses to the two types of stresses, no systematic pattern was observed regarding synergies or trade-offs under the two types of stresses. Therefore, we conclude that despite the similarities in physiological stress responses to ozone and blast, the tolerance to these stresses does not appear to be genetically linked in rice.

**Supplementary Information:**

The online version contains supplementary material available at 10.1007/s11356-022-19282-z.

## Introduction

Crops are exposed to both abiotic and biotic stresses in the field (Chojak-Koźniewska et al. 2018; Cohen and Leac 2019). Increasing tropospheric ozone concentrations and blast disease caused by *Magnaporthe oryzae* are two coinciding stresses affecting rice (*Oryza sativa* L.) yield worldwide (Singh et al. 2020; Ashrafuzzaman et al. 2021). The annual yield loss due to rice blast disease is estimated at 10–30% (Wilson and Talbot 2009; Ashkani et al. 2015; Sakulkoo et al. 2018), whereas ozone reduces global rice yield by an estimated 4.4% annually (Mills et al. 2018). Current approaches for testing and developing stress-tolerant rice varieties by applying either single biotic stress (e.g., bacteria, fungi, nematodes, insects, etc.) or abiotic stress (e.g., ozone, drought, salinity, submergence, etc.) may insufficiently account for synergies or trade-offs in tolerance mechanisms (Mittler and Blumwald 2010; Atkinson and Urwin 2012; Agathokleous et al. 2021). Therefore, the interactions of multiple abiotic and biotic stresses need to be considered in crop breeding.

Ozone is formed due to complex photochemical reactions of precursor gases such as nitrous oxide (NOx), carbon monoxide (CO), and volatile organic compounds (VOCs) in the presence of sunlight (Otero et al. 2021). Its concentration has been rising in the troposphere (Tarasick et al. 2019; Yang et al. 2020). In different growth stages, major crops such as maize, wheat, rice, and soybean, regularly face ozone stress with concentrations of ~60–100 ppb (Ainsworth 2017), which leads to total annual yield losses estimated at 200 million tons (Mills et al. 2018). Regionally, ozone stress leads to 10% of rice yield losses, which may exacerbate in the future with further increases in ozone levels in South and East Asia, especially in India and Bangladesh (Ainsworth 2008; Van Dingenen et al. 2009; Frei 2015; Mahmood et al. 2021). For example, with an increase of 1% in tropospheric ozone concentration in South Asian countries, rice productivity decreases by 2.3% (Mahmood et al. 2020).

Ozone diffuses into plants through the stomata during photosynthetic gas exchange and generates reactive oxygen species (ROS) in the apoplast (Vahisalu et al. 2010). Therefore, foliar necrotic symptoms can occur due to direct tissue damage caused by ROS or programmed cell death (PCD) induced by ROS (Kangasjärvi et al., 2005). Furthermore, as a response to ozone, plants restrict the stomatal opening, thus limiting the carbon dioxide uptake from the air reducing photosynthetic capacity (Frei 2015; Mahmood et al. 2020). Other critical physiological responses of crops to ozone include lipid peroxidation in cellular membranes, protein denaturation, pigment breakdown, and premature leaf senescence (Frei 2015; Ainsworth 2017), leading to reduced crop biomass, yield, and quality (Ashrafuzzaman et al. 2018; Emberson et al. 2018; Begum et al. 2020; Mahmood et al. 2020).

Blast disease is caused by the filamentous ascomycete’s fungus *Magnaporthe oryzae* and affects rice yield in rice-growing regions worldwide (Faivre-Rampant et al. 2013). The severity of rice blast disease depends on climate conditions: high relative humidity >90%, and temperature ranges from 24–30 °C can lead to an epidemic of dreaded blast diseases (Hensawang et al. 2017). The blast fungus can infect rice plants at all development stages resulting in leaf, node, neck, and panicle blast. Under favorable conditions, foliar infection is initiated by the attachment of a three-celled conidium of *M. oryzae* to the rice leaf cuticle (Wilson and Talbot 2009). About 4 to 5 days after infection (Boddy 2016), necrotrophic lesions appear on leaves, in which the fungus sporulates profusely, thus allowing the disease to spread rapidly to adjacent rice plants (Hamer et al. 1988; Talbot 2003). The outbreak of this disease is a threat to global food security, as rice-growing Asian and African countries can incur 60 to 100% yield loss under epidemic conditions of the blast (Kihoro et al. 2013). Annual global yield loss of rice due to blast is equivalent to the amount of rice required to feed 60 million people (Pennisi 2010).

Thus, these two stresses put food security at risk, as rice is the staple food crop for Asian countries (Frei 2015). Individual effects of tropospheric ozone and blast on rice yield and quality were well documented. Some studies have investigated the simultaneous fungal attack and ozone exposure in different plants (Wukasch and Hofstra 1977; Pazarlar et al. 2017). However, the combined effect of ozone and blast on rice was not previously evaluated, although blast overlaps with the peak ambient ozone concentrations in South and East Asia (Khush and Jena 2009; Frei 2015). Hur et al. (2002) found that rice blast conidia cultured under acute ozone showed reduced pathogenicity on rice plants grown in an ozone-free environment. The abundance of blast disease in areas where ambient ozone occurs at high levels may lead to interactions between the two factors in field-grown plants. However, no information is available on whether there is a synergy or a trade-off in tolerance or resistance against these different stresses in rice. In fact, ozone has been characterized as an abiotic elicitor of plant defense reactions (Sandermann et al. 1998). Based on many studies, the action of ozone is hormetic, which means that it enhances plant defense mechanisms and preconditions plants against other environmental challenges when not exceeding the toxicological threshold (Agathokleous et al. 2019). The common denominator of both stresses could be PCD, a characteristic plant response in ozone stress and pathogen infections (Heath 2000; Kangasjärvi et al. 2005). Disease resistance involves confining pathogens in dead cells by triggering PCD, thereby preventing their spread to the other tissues (Apel and Hirt 2004). On the other hand, ozone tolerance required avoidance of programmed cell death caused by ozone-derived apoplastic ROS (Ueda et al. 2015b). Therefore, this study aimed at evaluating different rice genotypes under the combined treatment of ozone and blast stress. Our specific research questions were (i) Does ozone exposure affect the plants’ responses to blast disease and vice versa? (ii) Are ozone and blast tolerance correlated in different rice genotypes, either positively or negatively? In order to address these questions, we conducted an experiment with nine diverse genotypes exposed to ozone and blast stress either alone or in combination.

## Materials and methods

### Plant materials and growth conditions

The experiment was conducted in a climate-controlled greenhouse from September 2020 to February 2021. Nine different rice genotypes were used in this experiment: (i) Nipponbare, an ozone sensitive Japanese *Japonica* rice variety (Jing et al. 2016); (ii) BRRI dhan28, an ozone sensitive and popular Bangladeshi *Indica* rice variety (Ashrafuzzaman et al. 2018); (iii) Binadhan-11, an ozone sensitive and Bangladeshi modern *Indica* rice variety (Ashrafuzzaman et al. 2018); (iv) IR64, an ozone sensitive (Ashrafuzzaman et al. 2018) and one of the world’s most widely grown *Indica* rice varieties, also known as blast-resistant (Sallaud et al. 2003); (v) Kasalath, a Bangladeshi *Aus* landrace which is the donor for ozone tolerant quantitative trait loci (Frei et al. 2008, 2010); (vi) L81, an ozone tolerant genotype carrying introgressions of two ozone tolerant quantitative trait loci from Kasalath in the background of Nipponbare (Wang et al. 2014); (vii) CO39, a blast-susceptible *Indica* rice genotype (Telebanco-Yanoria et al. 2011), (viii) Koshihikari, a blast-susceptible short-grain rice *Japonica* cultivar (Kobayashi et al. 2018); (ix) Kitaake, a model *Japonica* rice cultivar (Li et al. 2017). These seeds were collected from plants grown in a greenhouse at the University of Bonn, Germany that had no stress exposure.

Seeds were germinated at 30 °C in deionized water in the dark for 3 days (Ashrafuzzaman et al. 2018). The seedlings were then transferred to a mesh floating on solutions containing one-fourth strength Yoshida nutrient solution (pH 5.5) and placed under natural light in the greenhouse for 7 days (Yoshida et al. 1976). The pH was adjusted to 5.5.

A total of one hundred forty-four pots were filled with local clay-silt luvisol soil with 16% clay, 77% silt, 7% sand, 1.2% organic carbon, and pH 6.5. To ensure balanced nutrition, “NovaTec classic 12-8-16” (12% N, 8% P2O5, 16% K2O) was applied initially at the rate of 0.8 g/pot (Ueda et al. 2015a; Ashrafuzzaman et al. 2017). The same fertilizer dose was applied at the reproductive stage. For blast inoculation, two seedlings of each genotype at 10-d were sown into a 10-cm diameter pot. The pots were placed in trays filled with water from transplanting throughout the growing season. Supplementary lighting was provided in the greenhouse from 7 a.m. to 6 p.m. to ensure a minimum photosynthetic photon flux density (PPFD) of 300 μmol m^−2^ s^−1^. The minimum temperature of the greenhouse was set to 28/22 °C (day/night), and the average humidity was 53% (Ashrafuzzaman et al. 2018).

Four different treatments with four replicates were implemented: (a) control, (b) blast, (c) ozone, and (d) ozone and blast. In total, eight open-top chambers (length 1 m, width 1 m, height 1 m) were used for control (4 chambers) and ozone fumigation (4 chambers). In each chamber, there were two trays, and each tray accommodated nine pots of different genotypes (two plants per pot). Plants from only one tray in each chamber were infected with the blast.

### Growth of fungal pathogen, inoculum preparation, and inoculation of rice plants

*Magnaporthe oryzae* isolate Li1497 (1328) was used for blast inoculation. Isolate Li1497 was grown on potato dextrose agar (PDA) for 7 days and then sub-cultured on rice leaf agar (50 g fresh rice leaves, 15 g agar, 10 g soluble starch, 2 g yeast extract in 1000 ml water). The cultures were incubated under UV light (16/8 h day/night) at 25 °C for 14 days to induce sporulation. Conidia of *M. oryzae* were harvested by scraping off the mycelia using tap water with a drop of Tween 20 and 0.4% gelatin and then strained through a double layer of cheesecloth. Rice plants at the three-leaf stage (24 days old seedlings) were inoculated by spraying with conidial suspensions (10^5^ conidia/ml) using a hand sprayer, which is optimal for visible disease reactions (Li et al. 2014; Zhang et al. 2014; Deng et al. 2016; Chen et al. 2019; Chakraborty et al. 2020; Norvienyeku et al. 2021). The inoculated plants were kept in a dark, moist incubation chamber at 25 °C and >95% RH for 24 h and were subsequently taken back to the greenhouse. The other non-inoculated plants were also kept in a dark, moist chamber to ensure the same growth condition.

### Ozone treatment

Plants were exposed to an ozone treatment from 15 DAT (days after transplanting) to 137 DAT (until the end of growth season) in open-top chambers (OTC) (Ueda et al. 2015a). A custom-made ozone generator (UB 01; Gemke Technik GmbH, Ennepetal, Germany) was used to ensure an ozone concentration of 100 ppb for 7 h (9:00–16:00 h) every day. As input, dried air passing through silica gels was used, and the generated ozone was first percolated through the water to remove reactive gases other than ozone. Then ozone-enriched air was blown into the chambers and evenly distributed via perforated plastic pipes running above the plant canopy. The ozone output was regulated by an ozone monitor (K100 W; Dr. A. Kuntze GmbH, Meerbusch, Germany) and detected by an ozone sensor (GE 760 ozone; Dr. A. Kuntze GmbH, Meerbusch, Germany) placed inside the fumigation chambers. Besides, the ozone concentrations were continuously monitored in the different chambers with an independent handheld ozone monitor (series 500; Aeroqual Ltd. Auckland, New Zealand) at 5-min intervals. The average recorded ozone concentration was 103 ± 12 ppb (average ± standard error) in the ozone treatment, whereas the average concentration in control conditions was 22 ± 6 ppb. Control plants were exposed to ambient ozone concentrations, but in the control conditions, the ambient ozone concentrations were maintained below the damage threshold level (40 ppb) (Ashrafuzzaman et al. 2018). Ozone fumigation was continued for 123 days until all genotypes reached maturity.

### Assessment of leaf blast severity

Visual leaf blast symptoms were quantified as blast severity score (BSS) using a scoring scale ranging from 0 to 9 (Hensawang et al. 2017), which was assessed 11, 21, and 61 DAI (days after inoculation). Score classification of rice blast disease and disease severity level was as follows: no lesion observed (score 0, severity 0%), small brown specks of pin-point size, or larger brown specks without a sporulating center (score 1, severity 1%), small roundish to slightly elongated, necrotic gray spots, about 1–2 mm in diameter, with a distinct brown margin (score 3, severity 5%), necrotic gray spots about 1–2 mm, with a brown margin, typical blast lesions infecting 4–10% of the leaf area (score 5, severity 25%), necrotic gray lesion about 2–5 mm, with a yellow margin, typical blast lesions infecting 26–50% of the leaf area (score 7, severity 50%), the lesion expands more than 75% leaf area affected (score 9, severity 75%) (Hensawang et al. 2017).

### Evaluation of ozone-induced leaf symptoms

Visible leaf symptoms of ozone stress as leaf bronzing score (LBS) were assigned at 10, 20, and 60 DAO (days after ozone exposure) to two fully expanded leaves of each plant as previously described (Frei et al. 2008; Ueda et al. 2015a). The score ranged from 0 (no ozone-induced symptoms) to 10 (the whole leaf severely damaged).

### Spectral reflectance and stomatal conductance

Spectral reflectance measurements were taken using a Polypen RP410 instrument (Photon Systems Instruments, Drasov, Czech Republic) three times at 10, 20, and 60 DAO. Three points were measured from the second youngest fully expanded leaf of each plant, and the average of the three points was calculated. The following indices were determined: normalized difference vegetation index (NDVI) = (*R*_780_ – *R*_630_)/(*R*_780_ + *R*_630_) (Rouse et al. 1973); photochemical reflectance index (PRI) = (*R*_528_ − *R*_567_)/(*R*_528_ + *R*_567_) (Gamon et al. 1992); Lichtenthaler index 2 (Lic2) = *R*_440_/*R*_690_ (Lichtenthaler et al. 1996); and anthocyanin reflectance index 1 (ARI1) = 1/*R*_550_ – 1/*R*_700_ (Gitelson et al. 2001). Vegetation indices were selected based on significant differences between treatments and relatedness with ozone stress. Stomatal conductance measurements were performed at 20 DAO using a leaf porometer (model SC1, Decagon Devices, Pullman, WA). Two points were measured from each plant's second-youngest fully expanded leaf, and the average of the two points was calculated.

### Biomass and yield

Plants were harvested when all genotypes had reached maturity. During harvesting, plant height, tiller number, and panicle numbers were measured. Harvested plants were dried in the oven at 50 °C for 72 h, and other agronomic characteristics such as single plant weight, filled grain number, hundred kernel weight, grain yield, straw biomass, and harvest index were measured.

### Lipid peroxidation analysis

To evaluate the lipid peroxidation in different genotypes, malondialdehyde (MDA) content in the shoot was quantified from each treatment at 20 DAO. The samples were collected between 10:00 and 12:00 h, immediately frozen in liquid nitrogen, and stored at −80 °C until further analysis. The amount of MDA was measured as described previously (Hodges et al. 1999; Höller et al. 2014). Extraction was performed from approximately 100 mg of ground tissues with 1.5 mL of 0.1% (*w*/*v*) trichloroacetic acid (TCA). After ultrasonication for 5 min, samples were centrifuged at 4 °C, and 14,000 g for 15 min, and the supernatants were divided into two aliquots of 500 μL into 14 ml falcon-tube. These aliquots of the same extract were mixed with reaction solution I (background reference) that contained 0.01% (*w/v*) 2,6-di-tert-butyl-4-methylphenol (BHT) dissolved in 20% TCA (*w/v*), and reaction solution II additionally containing 0.65% 2-thiobarbituric acid (TBA), respectively. The mixture was then heated to 95 °C for 30 min, and the absorbance was measured at 440, 532, and 600 nm. Blank samples were also prepared with 0.1% (*w*/*v*) TCA solution instead of sample supernatant, and the absorbance was subtracted from each sample value.

### Statistical analysis

Analysis of variance (ANOVA) was performed by mixed model three-way ANOVA using the program R (R for Windows 3.5.1), packages nlme, and emmeans (R Core Team 2018). Ozone, blast, genotype, and their interactions were considered fixed effects, while chamber as a random effect. The mean comparison was performed by Tukey's test for post hoc adjustment, and *P*-values less than 0.05 were considered significant. Vegetation indices at 20 DAO, LBS at 20 DAO, BSS at 20 DAO, stomatal conductance at 20 DAO, MDA at 20 DAO, panicle number, filled grain number, straw biomass, and grain yield was used for the Pearson correlation matrix analysis.

## Results

### Differential visual symptoms in response to ozone and blast inoculation

After blast inoculation and exposure to ozone, plants were repeatedly phenotyped using the visual scoring scale; LBS for ozone and BSS for the blast. Visual symptoms did not occur in control plants but were only seen in plants exposed to ozone, blast, or combined treatment. Blast inoculated plants showed a significant average decrease in BSS under ozone fumigation. In contrast, blast inoculation did not significantly affect leaf bronzing score to ozone in all three sampling dates (Table 1).

**Table 1:** Descriptive statistics and ANOVA of physiological data under stress and control conditions

Traits	Date	LS means (treatment)								ANOVA results (Pr > F)						
		Control		Blast		Ozone		Ozone and blast								
		Mean	SD	Mean	SD	Mean	SD	Mean	SD	Bl	Oz	Ge	BlxGe	OzxGe	OzxBl	OzxBlxGe
LBS	10 DAO	n.d.	n.d.	n.d.	n.d.	5.28^a^	1.83	5.38^a^	1.96	n.d.	n.d.	<0.0001	0.923	n.d.	n.d.	n.d.
	20 DAO	n.d.	n.d.	n.d.	n.d.	5.75^a^	2.15	5.94^a^	2.14	n.d.	n.d.	<0.0001	0.634	n.d.	n.d.	n.d.
	60 DAO	n.d.	n.d.	n.d.	n.d.	5.42^a^	1.81	5.51^a^	1.91	n.d.	n.d.	<0.0001	0.751	n.d.	n.d.	n.d.
BSS	10 DAO	n.d.	n.d.	1.67^a^	1.88	n.d.	n.d.	1.44^b^	1.63	n.d.	n.d.	<0.0001	n.d.	<0.0001	n.d.	n.d.
	20 DAO	n.d.	n.d.	2.44^a^	2.54	n.d.	n.d.	1.89^b^	1.87	n.d.	n.d.	<0.0001	n.d.	<0.0001	n.d.	n.d.
	60 DAO	n.d.	n.d.	2.44^a^	2.54	n.d.	n.d.	1.89^b^	1.87	n.d.	n.d.	<0.0001	n.d.	<0.0001	n.d.	n.d.
NDVI	10 DAO	0.52^a^	0.05	0.50^a^	0.09	0.36^c^	0.10	0.41^b^	0.09	0.0816	0.0047	<0.0001	0.2447	<0.0001	0.0001	0.0009
	20 DAO	0.58^a^	0.06	0.42^b^	0.16	0.36^c^	0.13	0.35^c^	0.13	<0.0001	0.0001	<0.0001	0.0001	<0.0001	<0.0001	0.0002
	60 DAO	0.72^a^	0.03	0.72^a^	0.03	0.58^b^	0.09	0.57^b^	0.10	0.3998	0.0001	<0.0001	0.4164	<0.0001	0.5561	0.3209
PRI	10 DAO	0.01^a^	0.01	0.01^a^	0.02	−0.03^c^	0.03	−0.02^b^	0.03	0.0369	0.0006	<0.0001	0.1744	<0.0001	<0.0001	<0.0001
	20 DAO	0.02^a^	0.02	−0.02^b^	0.04	−0.04^c^	0.04	−0.04^c^	0.04	<0.0001	0.0001	<0.0001	0.0001	<0.0001	<0.0001	<0.0001
	60 DAO	0.04^a^	0.01	0.03^a^	0.01	−0.02^b^	0.03	−0.02^b^	0.03	0.9456	0.0001	<0.0001	0.8430	<0.0001	0.3986	0.6792
Lic2	10 DAO	0.64^b^	0.08	0.68^a^	0.10	0.52^d^	0.10	0.60^c^	0.11	<0.0001	0.0432	<0.0001	0.0080	<0.0001	0.0041	0.0003
	20 DAO	0.74^a^	0.11	0.64^b^	0.16	0.55^c^	0.14	0.55^c^	0.14	<0.0001	0.0010	<0.0001	0.0136	<0.0001	<0.0001	<0.0001
	60 DAO	0.92^a^	0.04	0.91^a^	0.05	0.68^b^	0.13	0.65^b^	0.14	0.0517	<0.0001	<0.0001	0.9211	<0.0001	0.2596	0.3756
ARI1	10DAO	−0.24^b^	0.18	−0.14^b^	0.36	0.39^a^	0.42	0.31^a^	0.44	0.7216	0.0024	<0.0001	0.2346	<0.0001	0.0015	<0.0001
	20 DAO	−0.26^c^	0.27	0.22^b^	0.61	0.53^a^	0.59	0.61^a^	0.59	<0.0001	0.0001	<0.0001	<0.0001	<0.0001	<0.0001	<0.0001
	60 DAO	−0.61^b^	0.15	−0.61^b^	0.16	0.36^a^	0.80	0.25^a^	0.65	0.0913	0.0001	<0.0001	0.6156	<0.0001	0.1479	0.5260
SC (mmol m^−2^ s^−1^)	20 DAO	418^a^	20.88	332^b^	15.72	265^c^	9.73	261^c^	10.07	<0.0001	<0.0001	<0.0001	<0.0001	<0.0001	<0.0001	<0.0001
MDA (nmol g^−1^) FW	20 DAO	8.61^c^	0.86	10.27^b^	1.67	13.08^a^	2.05	12.74^a^	1.78	0.0001	0.0002	<0.0001	0.0443	<0.0001	<0.0001	0.0003

Note: Mean values of all genotypes are shown. Different superscript letters following mean values within one row indicate significant differences at *p* < 0.05 by Tukey’s HSD test. *LS*, least square means; *SD*, standard deviation; *Bl*, blast; *Oz*, ozone; *Ge*, genotype; *DAO*, days after ozone exposure; *LBS*, leaf bronzing score; *BBS*, blast severity score; *n.d*., not determined; *NDVI*, normalized difference vegetation index; *PRI*, photochemical reflectance index; *Lic2*, Lichtenthaler index 2; *ARI1*, anthocyanin reflectance index 1; *SC*, stomatal conductance (mmol m^−2^ s^−1^); *MDA*, malondialdehyde (nmol g^−1^); *FW*, fresh weight

LBS and BSS exhibited highly significant genotypic differences. The most visible ozone damage was seen in CO39, followed by Binadhan-11, IR64, BRRI dhan28, Koshihikari, Nipponbare, Kitaake, and the least symptoms in Kasalath and L81 (Supplementary Table 1). BSS was highest in CO39, followed by Koshihikari, Nipponbare, BRRI dhan28, IR64, and Kasalath. No blast symptoms were observed in Binadhan-11 and Kitaake. Under combined ozone and blast treatment, CO39 and L81 showed a decreased BSS, whereas the BSS did not change for other genotypes (Supplementary Table 1). Overall, the visual symptom assessment demonstrated that ozone exposure rather reduced blast severity; in contrast, blast disease did not significantly affect ozone sensitivity.

### Spectral reflectance indices

When averaged across all genotypes, a significant response of vegetation indices was seen due to ozone, blast, and ozone and blast treatment. Comparing treatment responses on the individual sampling days, significant effects of blast treatment occurred at 20 DAO for all the indices. In addition, a significant effect caused by blast on PRI and Lic2 was also seen on DAO 10. Blast by genotype interaction was primarily seen at 20 DAO. In contrast, vegetation indices significantly responded to ozone in all three sampling dates. In addition, significant ozone by genotype interaction was observed. Significant interactions between ozone and blast and among ozone, blast, and genotype were seen at 10 and 20 DAO for all vegetation indices (Table 1). All the indices demonstrated highly significant genotypic differences.

**Fig. 1:**
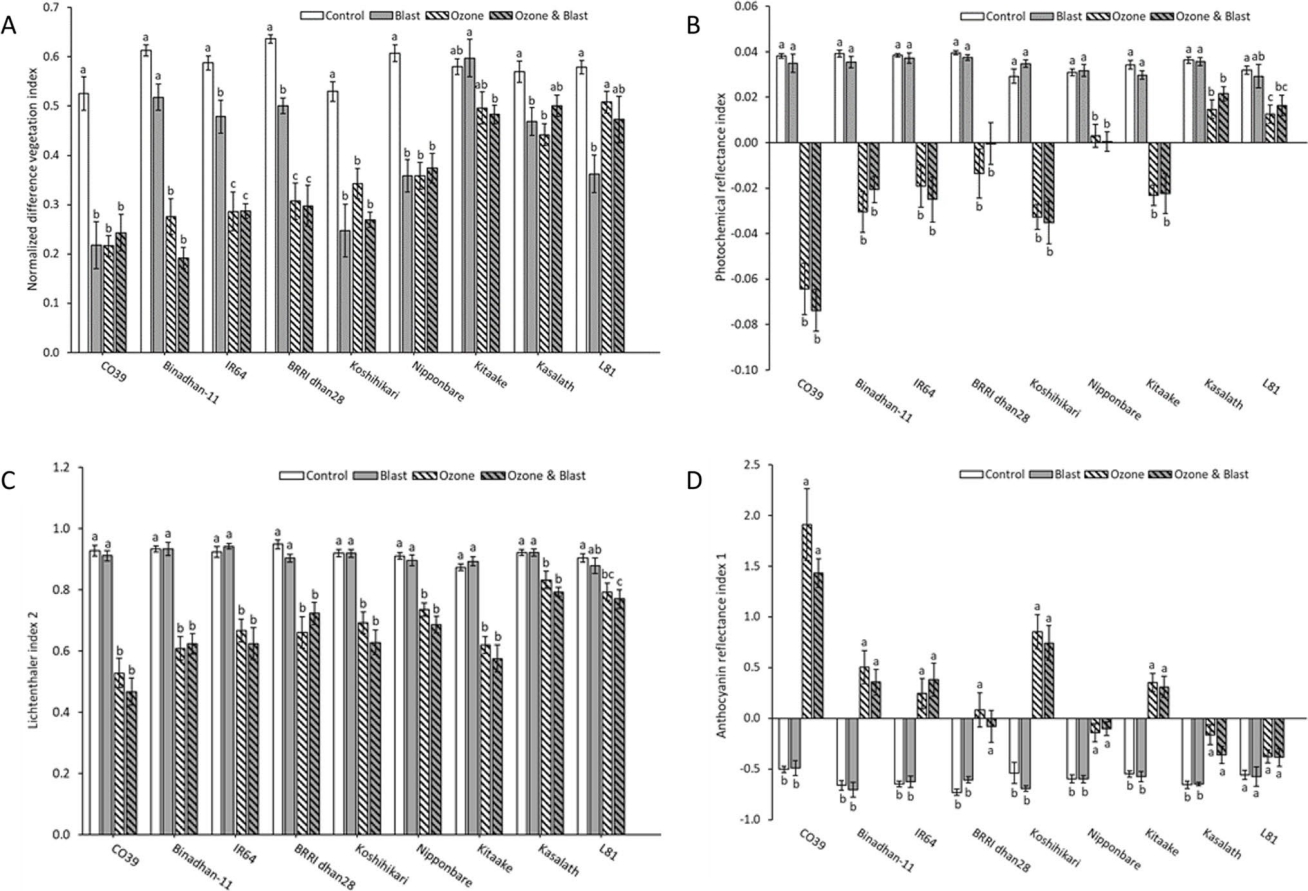
Vegetation indices at 20 DAO based on the reflectance spectra of nine rice genotypes exposed to ozone, blast, ozone and blast, or control conditions. *Y*-axis represents different indices and bars indicate the mean value ± standard errors (*n* = 8), *X*-axis represents different rice genotypes. Letters above the bars indicate pair-wise comparison (*P* < 0.05) within the genotype (mean values not sharing the same letter are significantly different)

Values for NDVI, a proxy for leaf greenness (chlorophyll content), were significantly lower in blast-infected plants than in the control at 20 DAO. Leaf greenness was significantly lower in ozone-affected plants than in the blast or control treatment on all three sampling days. Interestingly, double stress, i.e., ozone and blast, did not significantly reduce leaf greenness compared to ozone stress only (Table 1). Comparing individual genotypes at 20 DAO, leaf greenness was not affected in blast-exposed Binadhan-11 and Kitaake. However, all ozone-treated plants showed a significant decrease in leaf greenness compared to control except for highly ozone tolerant L81. For combined ozone and blast stress, none of the plants showed a significant change in leaf chlorophyll content compared to ozone stress only (Fig. 1A).

PRI estimates the photosynthetic light use efficiency and showed significantly decreased values in blast-affected plants compared to control at 20 DAO. In all three sampling dates, ozone treatment induced a significant reduction in PRI compared to blast or control. No significant difference was observed between ozone and ozone and blast except for 10 DAO (Table 1). When comparing genotypes at 20 DAO, no significant differences were seen between ozone and ozone and blast. All the genotypes under ozone and ozone and blast treatments showed a significant reduction in PRI compared to control and blast, except for L81, which showed no significant differences between blast and ozone and blast treatment (Fig. 1B).

Vegetation index Lic2 represents the carotenoid to chlorophyll pigment ratio, which tends to decrease under stress conditions. A significant decrease in Lic2 in ozone stress compared to control or blast was observed. At 60 DAO, Lic2 did not significantly change in the blast compared to the control, and no significant response in Lic2 was observed in the ozone and blast compared to ozone except for 10 DAO (Table 1). None of the genotypes showed significant responses to blast compared to control and to ozone compared to ozone and blast for Lic2 (Fig. 1C). In contrast, we observed a significant decrease in Lic2 in ozone and ozone and blast compared to control or blast except for L81, which showed nonsignificant responses between blast and ozone (Fig. 1C).

ARI1 represents anthocyanin level in plants and was significantly higher in the blast treatment compared to control at 20 DAO. In addition, ARI1 was higher in ozone and ozone and blast compared to control or blast, while no significant difference was seen between ozone and ozone and blast (Table 1). Ozone-tolerant genotype L81 did not show any significant variation for ARI1 among all treatments. The highest ARI1 was seen in CO39 under ozone stress (Fig. 1D), which is highly susceptible to blast or ozone. However, there were no significant differences for ARI1 between control and blast and ozone and ozone and blast in any genotypes (Fig. 1D).

In summary, vegetation indices were generally affected under ozone fumigation. On the other hand, in double stress, vegetation indices were not significantly different from those in ozone stress but rather worse than in the blast treatment. Moreover, prolonged ozone fumigation increased the adverse effect, whereas blast severity did not increase in plants after a certain period.

### Physiological characteristics

**Fig. 2:**
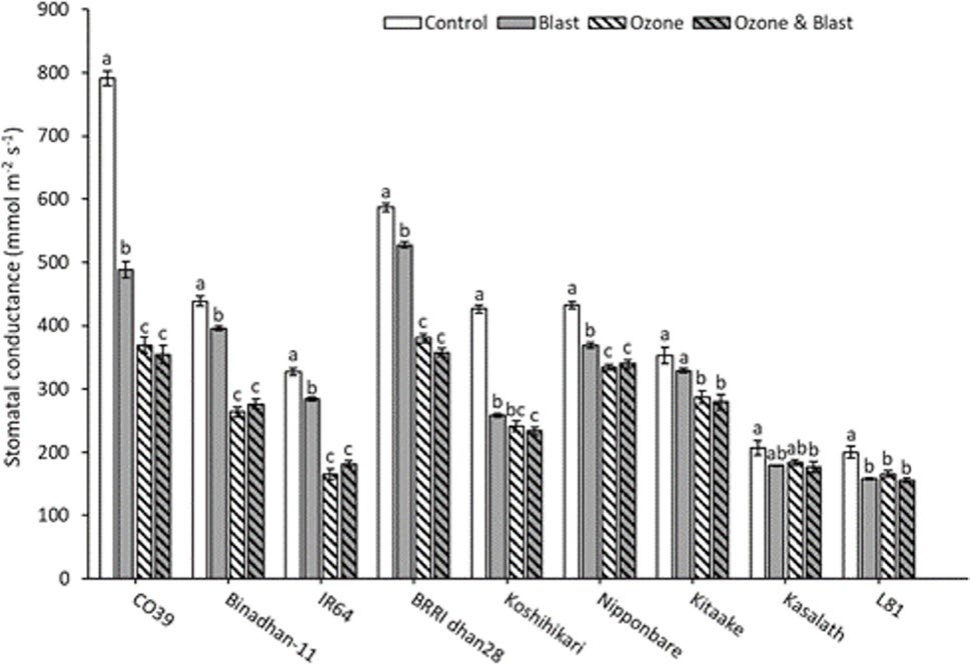
Stomatal conductance (mmol m^−2^ s^−1^) at 20 DAO of nine rice genotypes exposed to ozone, blast, ozone and blast, or control conditions. Bars indicate the mean value ± standard errors (*n* = 8). Letters above the bars indicate pair-wise comparison (*P* < 0.05) within the genotype (mean values not sharing the same letter are significantly different)

As a proxy for photosynthetic gas exchange, stomatal conductance was measured at 20 DAO. Individual and combined treatment effects and highly significant genotypic differences were seen. Compared to control, stomatal conductance was significantly lower in the blast, ozone, and ozone and blast. However, there was no significant difference between ozone and ozone and blast (Table 1). Significantly reduced stomatal conductance was observed in all the genotypes except for Kitaake and Kasalath in blast compared to control. Stomatal conductance was not lower in ozone and blast than in ozone, and in most cases, both were significantly lower than control or blast. Ozone-tolerant L81 showed a significant difference between control and other treatments, and Kasalath showed a significant difference between control and ozone and blast (Fig. 2).

**Fig. 3:**
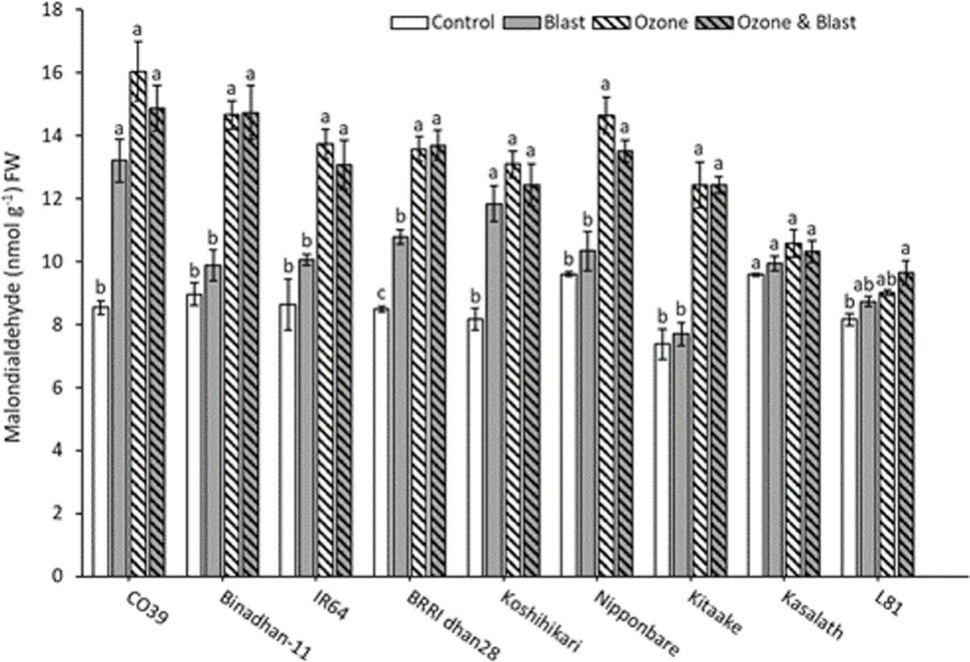
Malondialdehyde (MDA) concentrations at 20 DAO in leaves of nine rice genotypes exposed to ozone, blast, ozone and blast, or control conditions. Bars indicate mean value ± standard errors (*n* = 3), fresh weight (FW). Letters above the bars indicate pair-wise comparison (*P* < 0.05) within the genotype (mean values not sharing the same letter are significantly different)

MDA concentration was also measured from the plants harvested at 20 DAO to quantify lipid peroxidation as an indicator of oxidative stress. Averaged over all genotypes, significant increases of MDA occurred due to individual and combined stress treatments. Shoot MDA concentration was significantly higher in ozone than in control or blast, but there was no significant difference between ozone and ozone and blast. In addition, blast led to significantly higher MDA concentration than control (Table 1). Kasalath did not show any significant increase in lipid peroxidation in any of the treatments, while L81 showed significantly elevated MDA only in ozone and blast compared to control (Fig. 3). Ozone and blast susceptible CO39 and Koshihikari showed significantly higher MDA concentration in the blast, ozone, and ozone and blast compared to control. Other genotypes, i.e., Binadhan-11, IR64, BRRI dhan28, Nipponbare, and Kitaake, did not show significant differences in MDA concentration between control and blast. The same trend was observed for ozone and ozone and blast; however, ozone and ozone and blast showed significantly higher MDA than control or blast for those genotypes. Generally, our results suggested that ozone caused much higher oxidative stress than blast (Fig. 3).

In summary, photosynthetic gas exchange and lipid peroxidation were significantly affected by individual or combined stress, but no escalation occurred due to combined ozone and blast treatment.

### Yield components

Several yield components were determined to reflect both straw and grain yields. Six yield components such as panicle number, single plant weight (g), filled grain number, grain yield (g), straw biomass (g), and harvest index showed significant treatment effects due to the decline in the ozone treatment (Table 2). In addition, four traits such as panicle number, filled grain number, grain yield (g), and harvest index showed a considerable blast effect (Table 2). There was a significant interaction for ozone by genotype for all traits except for plant height, but no interaction was identified for a blast by genotype (Table 2). In addition, ozone by blast interaction was observed only for filled grain number, grain yield, and harvest index, but no interaction was seen for ozone, blast, and genotype (Table 2). Compared to the control, the average grain yield loss due to blast, ozone, and ozone and blast across all genotypes was around 17%, 37%, and 41%, respectively. However, the difference in yield loss between ozone and blast and ozone was statistically insignificant (*P* = 0.6568). Compared to the blast treatment and control, significant straw biomass reduction was observed in ozone and ozone and blast treatment (Table 2).

**Table 2:** Descriptive statistics and ANOVA results for phenotypic traits under stress and control conditions

Traits	LS means (treatment)								ANOVA results (Pr > F)						
	Control		Blast		Ozone		Ozone and blast								
	Mean	SD	Mean	SD	Mean	SD	Mean	SD	Bl	Oz	Ge	BlxGe	OzxGe	OzxBl	OzxBlxGe
Plant height (cm)	81.17^a^	19.20	82.08^a^	20.07	73.42^b^	20.98	74.44^b^	21.53	0.3132	0.0032	<0.0001	0.8742	0.1992	0.9539	0.8814
Tiller number	3.81^a^	1.26	3.47^ab^	1.13	3.36^ab^	0.93	3.17^b^	0.91	0.0287	0.0708	<0.0001	0.9849	0.0036	0.5605	0.9801
Panicle number	3.44^a^	1.23	2.92^b^	1.11	2.64^b^	0.93	2.56^b^	0.88	0.0100	0.0242	<0.0001	0.9554	0.0042	0.0592	0.9709
Single plant weight (g)	6.87^a^	3.48	6.36^a^	3.28	4.86^b^	3.12	4.88^b^	3.49	0.1197	<0.0001	<0.0001	0.8096	<0.0001	0.0876	0.1387
Filled grain number	133^a^	82	110^b^	75	82^c^	67	79^c^	59	0.0001	0.0001	<0.0001	0.2465	<0.0001	0.0040	0.6046
Hundred kernel weight (g)	1.96^a^	0.19	1.97^a^	0.22	2.01^a^	0.20	1.99^a^	0.20	0.6419	0.1655	<0.0001	0.7991	0.0014	0.5970	0.7856
Grain yield (g)	2.54^a^	1.37	2.09^b^	1.29	1.58^c^	1.11	1.49^c^	0.95	0.0001	0.0001	<0.0001	0.2160	<0.0001	0.0063	0.5559
Straw biomass (g)	4.33^a^	2.26	4.27^a^	2.14	3.29^b^	2.08	3.39^b^	2.60	0.8558	0.0003	<0.0001	0.6109	0.0037	0.5399	0.1321
Harvest index	0.37^a^	0.07	0.32^b^	0.07	0.33^b^	0.07	0.32^b^	0.08	0.0028	0.2134	<0.0001	0.6770	0.0774	0.0241	0.6064

Note: Mean values per individual plant of all genotypes are shown. *SD*, standard deviation; *LS means*, least square means; *Bl*, blast; *Oz*, ozone. LS mean values not sharing the same superscript letter are differ significantly from each other at *P* < 0.05 by Tukey HSD post hoc comparison

**Fig. 4:**
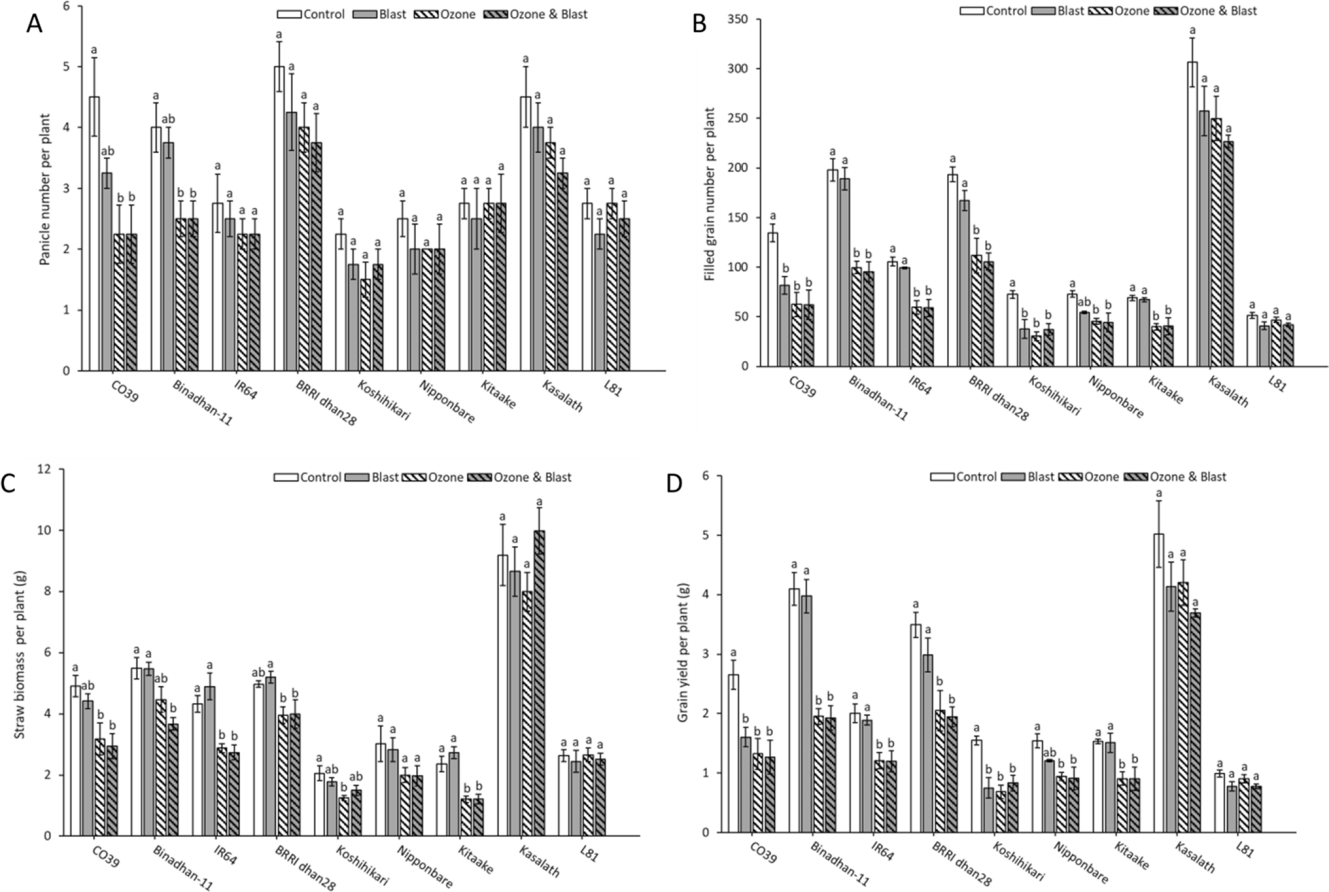
Yields and yield components of three different rice genotypes exposed to four different treatments of nine rice genotypes exposed to ozone, blast, ozone and blast, or control conditions. Bars indicate mean value ± standard errors (*n* = 4). Letters above the bars indicate pair-wise comparison (*P* < 0.05) within the genotype (mean values not sharing the same letter are significantly different)

All the harvest fractions exhibited highly significant genotypic differences (Table 2). Panicle number per plant was significantly reduced in CO39 and Binadhan-11 for ozone and ozone and blast compared to control; while other genotypes did not show any significant responses (Fig. 4A). The filled grain number was not significantly affected in the ozone tolerant genotypes Kasalath and L81 due to ozone or blast or ozone and blast (Fig. 4B). In contrast, a significant reduction in filled grain number was observed in CO39 and Koshihikari due to blast, ozone, and ozone and blast (Fig. 4B). Straw biomass was significantly reduced in most genotypes in ozone and ozone and blast compared to control, except for Nipponbare, L81, and Kasalath, which did not show a significant reduction (Fig. 4C). There was no escalation due to combined ozone and blast treatment compared to ozone in any genotype (Fig. 4C). Compared to control, a significant grain yield loss due to blast was seen only in CO39 and Koshihikari (Fig. 4D). However, most genotypes, except Kasalath and L81, showed a significantly reduced yield in ozone and ozone and blast-affected plants compared to control or blast. The combined ozone and blast treatment did not exacerbate yield loss in any genotype compared to individual ozone or blast treatment (Fig. 4D).

In conclusion, yield components were highly affected, mainly due to the negative effects of ozone on the filled grain number (Table 2).

### Correlations between traits

**Fig. 5:**
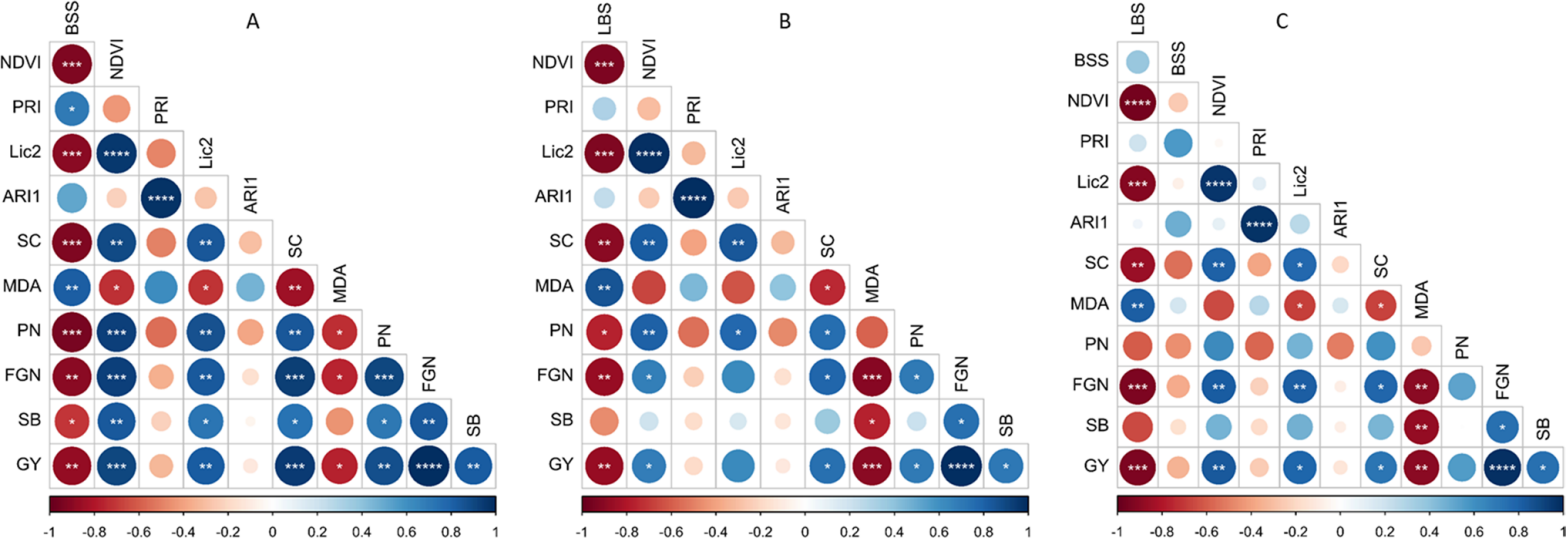
Pearson correlation matrix for phenotypic traits of rice genotypes exposed to blast (**A**), ozone (**B**), and ozone and blast (**C**). Asterisk indicates statistically significant correlation at *p < 0.05; ** *p* < 0.01; *p* < 0.001; ***; *p* < 0.0001****. BBS, blast severity score; LBS, leaf bronzing score; NDVI, normalized difference vegetation index; PRI, photochemical reflectance index; Lic2, Lichtenthaler index 2; ARI1, anthocyanin reflectance index 1; SC, stomatal conductance (mmol m^−2^ s^−1^); MDA malondialdehyde (nmol g^−1^); FW, fresh weight; PN, panicle number; FGN, filled grain number; SB, straw biomass (g); GY, grain yield (g). Relative values (ratio of value for plants grown under stress conditions relative to the control condition) were used except for BSS and LBS (*n* = 9)

We conducted a correlation analysis (Fig. 5) to analyze how different traits were interrelated within the three stress treatments (ozone, blast, and ozone and blast). For this analysis, we used relative values (value in the stress treatment/value in the control). The strongest correlations were seen in auto-correlated traits, i.e., between different vegetation indices or between different yield components. BSS showed a strong correlation with almost all of the traits within the blast treatment, and LBS in ozone treatment also significantly correlated with most of the traits. However, no significant correlation was observed for BSS within ozone and blast treatment, where LBS was significantly associated with most other traits (Fig. 5C). Notably, grain yield was significantly correlated with most traits when plants were exposed to individual treatment, i.e., ozone or blast. In the combined ozone and blast treatment, the strongest correlation was identified between grain yield and LBS rather than BSS (Fig. 5C). These data demonstrated that in combined ozone and blast treatment, ozone was the dominating stress for plants compared to blast.

## Discussion

### Interactions between ozone and blast stress

The first objective of this study was to explore interactions between ozone and blast stress on rice plants. Notably, many of the traits measured in this study demonstrated significant interactions between ozone and blast treatment (Tables 1 and 2). In order to quantify stress symptoms, we employed visual scoring scales. LBS as a measure for visible ozone damage (Ueda et al. 2015a,b; Ashrafuzzaman et al. 2017; Begum et al. 2020) and BSS as a measure for blast severity (Challagulla et al. 2015; Hensawang et al. 2017; Devi et al. 2020) have repeatedly been used in previous studies and are thus well established. In this experiment, under combined ozone and blast treatment, ozone exposure reduced blast severity (Table 1, Supplementary Table 1). Symptoms of oxidative stress triggered by ozone appeared as chlorosis and brown spots on the leaves, while diamond-shaped light tan lesions with necrotic borders (Supplementary Fig. 1) characterized blast symptoms. The differential appearance of LBS and BSS helped us to distinguish between ozone and blast injury in combined ozone and blast treatment. A previous report showed that rice blast fungus infection potential was inhibited by 200 ppb of acute ozone exposure for 3 days (Hur et al. 2002). However, in that experiment, they grew the blast conidia under ozone exposure and then inoculated plants which had not been exposed to ozone. Thus, they did not investigate plant reactions to single or combined stresses. When used at appropriate concentrations, ozone could trigger defense against pathogens (Pazarlar et al. 2017), as ozone generates ROS, which forms part of the primary defense mechanism in plants against pathogens (Torres et al. 2006; Huang et al. 2019). Our data suggested that long-term chronic ozone fumigation at 100 ppb did not favor the environment for blast conidia growth and infection. As a hemibiotrophic (Park et al. 2009; Fernandez and Orth 2018) fungal pathogen, *M. oryzae* requires living cells at the initial period (biotrophy). Thus, their feeding may be inhibited by ozone-induced leaf senescence and cell death (Violini 1995). Inside the plant, ozone-induced ROS may accelerate defense-like responses, including cell wall strengthening (e.g., through lignification) and induction of pathogen-associated defense genes (Sandermann et al. 1998; Fiscus et al. 2005). Some other biotrophic fungal pathogens also showed decreased disease severity under ozone fumigation, e.g., powdery mildew in barley (Mikkelsen et al., 2015), in wheat (Pazarlar et al. 2017), and cucumber (Khan and Khan 1999). However, young wheat plants showed a severe powdery mildew (biotrophic) attack when exposed to 80 to 160 ppb of ozone, while at a concentration of 240 ppb, powdery mildew attack was significantly reduced. In that study, very high ozone concentration enhanced the premature senescence of the wheat leaf, which inhibited the powdery mildew growth (Tiedemann 1992).

We employed a set of vegetation indices to estimate ozone and blast effects on foliar pigments at individual plant levels through non-destructive measurements (Sims and Gamon 2002; Meroni et al. 2009; López López et al., 2016). For different host-pathogen interactions, reductions in pigment concentrations are the most notable adverse effects resulting from pathogen infection (Lichtenthaler and Miehé 1997; Baker 2008). Apart from the commonly used NDVI, ozone responsive vegetation index Lic2 (Begum et al. 2020) was significantly positively correlated with grain yield in the blast and ozone and blast treatment. In many stressful situations, chlorophyll degrades faster than carotenoids (Penuelas et al. 1995; Liu et al. 2011), as reflected in Lic2. However, in the combined stress, the additional blast infection did not escalate the damaging effect.

One possible explanation for the mitigating effect of ozone on blast infection could be phytoalexin-type cellular compounds (Skarby and Pell 1979). The chemical substance phytoalexin inhibits the fungus development and is formed or activated only when the host plants contact the parasite (Harborne 1993). However, ozone resembles fungal elicitors, and phytoalexins were induced by ozone in soybean (Keen and Taylor 1975), pine needles (Sandermann 1996), and in grapevine (Schubert et al. 1997). Rice infected with *M. oryzae* showed resistant disease reactions probably through activation of ROS and phytoalexin production (Yang et al. 2017). In addition, ozone activates salicylic acid-dependent signaling pathways previously shown to be associated with the activation of pathogen defense reactions (Sharma et al. 1996; Rao and Davis 1999). In tobacco, the ozone-induced salicylic acid signaling pathway increased tolerance towards the tobacco mosaic virus (Yalpani et al., 1994).

Regarding the grain yield, a significant decline was seen in ozone and ozone and blast compared to blast or control. However, the most blast susceptible CO39 and Koshihikari contributed to a likewise significant yield loss in the blast treatment compared to control. In our study, season-long high ozone (103 ppb) treatment caused a 37% yield loss in rice (Table 2). For comparison, Ashrafuzzaman et al. (2017) reported grain yield losses in rice exceeding 26% after season-long ozone fumigation with an average ozone concentration of 77 ppb. Yield loss for the blast was around 17%, but in the combined ozone and blast, these yield losses from individual stresses did not simply sum up, but were only slightly and nonsignificantly higher than in the ozone alone treatment. In a previous study, the fungal disease powdery mildew combined with 100 ppb ozone did also not exacerbate yield loss in cucumber (Khan and Khan 1999). However, the reported yield loss due to blast is higher than for ozone (Mills et al. 2018; Sakulkoo et al. 2018), which is the opposite of our results. We exposed the plants to ozone for an entire season with a relatively high average concentration of 103 ppb ozone. Furthermore, only two out of nine genotypes were ozone tolerant in our experiment. Also, the genotypes used in this study showed no significant yield loss due to blast except for susceptible CO39 and Koshihikari. These factors may have resulted in higher yield losses due to ozone than due to blast.

These data together answered the first question of this study that ozone exposure does not increase the sensitivity of plants to blast; rather, it mitigated the formation of visible blast symptoms, and blast inoculation did not aggravate ozone sensitivity. However, in-depth physiological or genetic causes need to be explored in further studies.

### Contrasting genotypic response to blast disease and ozone

The second objective of this study was to explore whether ozone and blast tolerance are positively or negatively correlated in different rice genotypes. Judged by visual injury, some of the genotypes showed both ozone and blast susceptibility (CO39, Koshihikari, Nipponbare). The ozone-tolerant Kasalath showed 1% BSS (Supplementary table 1), which is considered a resistant reaction to blast inoculation (Hensawang et al. 2017; Xiao et al. 2017), whereas the ozone tolerant L81 (derived from Kasalath as one of its parents) showed blast susceptibility (Supplementary Table 1). It is possible that blast sensitivity in L81 was inherited from its second parent Nipponbare (Wang et al. 2014), which showed a similar level of blast sensitivity. In contrast, Kasalath showed a broad spectrum of resistance reactions against standard differential blast isolates from the Philippines and Japan in a previous study (Ebitani et al. 2011). Kasalath is also considered a donor for blast resistance QTL (Hayasaka et al. 1995; Takehisa et al. 2009). On the other hand, ozone susceptible Binadhan-11, Kitaake, IR64, and BRRI dhan28 showed blast resistance. Similar to visual injury, differential ozone or blast tolerance or susceptibility were also represented by vegetation indices and other physiological traits such as stomatal conductance and lipid peroxidation. Yield and yield components did not demonstrate any additive or interactive effect regarding blast or ozone tolerance (Table 2, Fig. 4). In addition, LBS and BSS were not significantly correlated in combined stress (Fig. 5C).

Ozone can induce plant-signaling cascades similar to a pathogen response, ultimately leading to PCD (Sandermann et al. 1998; Kangasjärvi et al., 2005). Moreover, PCD is an essential pathway of pathogen response in plant leaves (Huysmans et al. 2017), which is involved in the formation of ozone stress symptoms (Ueda et al. 2015a, b). Therefore, balancing the interplay of redox homeostasis and PCD pathways is essential for simultaneous ozone and pathogen tolerant breeding (Mills et al. 2018). In our study Kasalath, the donor for both ozone and blast tolerant QTL did not show any apparent conflict between the ozone and blast tolerance in combined ozone and blast stress. This genotype was resistant to both blast and ozone. In a recent study targeting ozone tolerance and fungal resistance breeding, Mashaheet et al. (2020) tested eight key rust-susceptible wheat genotypes for ozone tolerance and found differential responses. For example, bread wheat genotypes Thatcher and LMPG 6 showed severe sensitivity to ozone, whereas Chinese Spring showed tolerance for ozone-induced visible symptoms and biomass production. Taken together, our data suggest that despite the partly overlapping physiological responses to ozone and blast disease and the interactive effects of these stresses on rice plants (Tables 1 and 2), tolerance or resistance to these stress factors are genetically independent traits. Thus, we can assume that breeding for tolerance against one trait would not necessarily compromise the other trait.

## Conclusion

Our data suggest that chronic ozone exposure slightly mitigated blast severity, while vice versa, no significant effect occurred. Moreover, the combined stress treatment did not lead to an additive escalation of stress intensity. Regarding the tolerance of different genotypes to the different individual or combined stresses, we did not observe any systematic synergy or trade off. Therefore, tolerance to one of these stresses may not compromise the tolerance against the other stress in rice.

## Supplementary information

Supplementary Table 1 (DOCX 16 kb)

Supplementary Fig. 1 (DOCX 276 kb)
