# Supplementary material for: Interactive effects of tropospheric ozone and blast disease (Magnaporthe oryzae) on different rice genotypes
Source: Environ Sci Pollut Res Int. 2022 Feb 24;29(32):48893–907. doi: 10.1007/s11356-022-19282-z (PMC9252976; doi:10.1007/s11356-022-19282-z)
Supplement: Supplementary file 1 — (DOCX 16 kb) [file 11356_2022_19282_MOESM1_ESM.docx]

Environmental Science and Pollution Research

Interactive effects of tropospheric ozone and blast disease (*Magnaporthe oryzae*) on different rice genotypes

Muhammad Shahedul Alam^1^, Angeline Wanjiku Maina^2^, Yanru Feng^1,3^, Lin-Bo Wu^1^, Michael Frei^1*^

^1^ Department of Agronomy and Crop Physiology, Institute for Agronomy and Plant Breeding, Justus-Liebig University Giessen, 35390 Giessen, Germany

^2^ INRES Plant Pathology, University of Bonn, Germany

^3^ Institute for Crop Science and Resource Conservation (INRES), Crop Science, University of Bonn, 53115 Bonn, Germany

* Corresponding author

Email: michael.frei@agrar.uni-giessen.de

Tel. +49 641 9937430

Supplementary Table 1 Categorizing genotypes based on average leaf bronzing score (LBS) and Blast severity score (BSS)

| Genotypes | Leaf bronzing score (LBS) | | Genotype | Blast severity score (BSS) | |
| --- | --- | --- | --- | --- | --- |
|  | Ozone | Ozone & Blast |  | Blast | Ozone & Blast |
| CO 39 | 8.1 | 8.3 | CO 39 | 8 | 5 |
| Binadhan-11 | 7.5 | 8.1 | Koshihikari | 5 | 5 |
| IR 64 | 7.3 | 7.0 | L 81 | 3 | 1 |
| BRRI dhan28 | 7.0 | 7.1 | Nipponbare | 3 | 3 |
| Koshihikari | 6.3 | 6.9 | BRRI dhan28 | 1 | 1 |
| Nipponbare | 6.0 | 6.0 | IR 64 | 1 | 1 |
| Kitaake | 4.4 | 4.5 | Kasalath | 1 | 1 |
| Kasalath | 2.8 | 2.6 | Binadhan-11 | 0 | 0 |
| L 81 | 2.5 | 3.0 | Kitaake | 0 | 0 |
