# Supplementary material for: Interactive effects of tropospheric ozone and blast disease (Magnaporthe oryzae) on different rice genotypes
Source: Environ Sci Pollut Res Int. 2022 Feb 24;29(32):48893–907. doi: 10.1007/s11356-022-19282-z (PMC9252976; doi:10.1007/s11356-022-19282-z)
Supplement: Supplementary file 2 — (DOCX 276 kb) [file 11356_2022_19282_MOESM2_ESM.docx]

Environmental Science and Pollution Research

Interactive effects of tropospheric ozone and blast disease (*Magnaporthe oryzae*) on different rice genotypes

Muhammad Shahedul Alam^1^, Angeline Wanjiku Maina^2^, Yanru Feng^1,3^, Lin-Bo Wu^1^, Michael Frei^1*^

^1^ Department of Agronomy and Crop Physiology, Institute for Agronomy and Plant Breeding, Justus-Liebig University Giessen, 35390 Giessen, Germany

^2^ INRES Plant Pathology, University of Bonn, Germany

^3^ Institute for Crop Science and Resource Conservation (INRES), Crop Science, University of Bonn, 53115 Bonn, Germany

* Corresponding author

Email: michael.frei@agrar.uni-giessen.de

Tel. +49 641 9937430


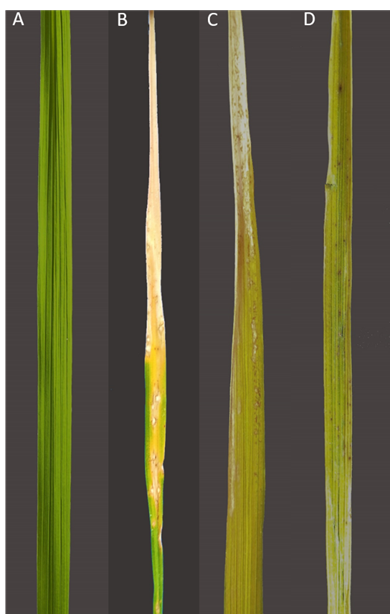


Supplementary Fig. 1 Differential visual injury by plant, (A) Control, (B) Blast, (C) Ozone, and (D) Ozone and Blast in combined ozone & Blast stress.
